# Open Distal Femoral Physeal Fracture in a 6-Year-Old Child Complicated by Growth Arrest and Limb-Length Discrepancy: A Case Report

**DOI:** 10.3390/children13060726

**Published:** 2026-05-23

**Authors:** Eglė Jauniškytė, Giedrė Žulpaitė, Jolanta Labanauskienė

**Affiliations:** 1 Faculty of Medicine, Vilnius University, M. K. Ciurlionio 21, 03101 Vilnius, Lithuania; 2 Department of Physiology, Biochemistry, Microbiology and Laboratory Medicine, Institute of Biomedical Sciences, Faculty of Medicine, Vilnius University, M. K. Ciurlionio 21, 03101 Vilnius, Lithuania; 3 Clinic of Children’s Diseases, Faculty of Medicine, Vilnius University, M. K. Ciurlionio 21, 03101 Vilnius, Lithuania

**Keywords:** distal femoral physeal fracture, Salter–Harris I, pediatric trauma, premature physeal closure, limb-length discrepancy, epiphysiodesis

## Abstract

Background/Objectives: Distal femoral physeal fractures are rare and particularly uncommon in very young patients, as they typically require a significant amount of kinetic energy. They carry a high risk of premature physeal closure and later growth disturbance. We aimed to describe the management and long-term outcome of an open distal femoral physeal fracture in a 6-year-old child. Methods: We report a previously healthy 6-year-old child sustained an open distal femoral physeal fracture in an electric scooter–motor vehicle collision. Emergency treatment included trauma assessment, resuscitation, intravenous cefazolin, urgent irrigation and debridement, open reduction, crossed smooth Kirschner-wire fixation, and immobilization. Long-term follow-up included growth prediction using the multiplier method. Results: The injury was classified intraoperatively as a Salter–Harris type I distal femoral physeal fracture. Despite timely surgical treatment, progressive limb-length discrepancy developed, increasing from 1.3 cm at 10 months to 6.5 cm over 5 years. Growth prediction estimated a final discrepancy of 7.32 cm at skeletal maturity, and contralateral distal femoral epiphysiodesis was performed. The literature confirms that displaced high-energy distal femoral physeal injuries in younger children carry a substantial risk of premature physeal closure and later corrective surgery. Conclusions: Open high-energy distal femoral physeal fractures in young children are limb-growth-threatening injuries. This case demonstrates that satisfactory initial fracture management does not eliminate the risk of later premature physeal closure, and that clinically important discrepancy evolves gradually over several years. Long-term follow-up and growth prediction are essential to guide timely corrective treatment to minimize the leg-length discrepancy in bone maturity.

## 1. Introduction

Distal femoral physeal fractures are rare in children, accounting for approximately 0.3–1.4% of pediatric fractures, but they are associated with a high risk of premature physeal closure and subsequent growth disturbance [1,2,3]. Because the distal femoral physis contributes significantly to longitudinal growth of the lower limb, injuries at this site may result in limb-length discrepancy and angular deformity, particularly after high-energy trauma [3,4]. The risk of growth arrest has been reported to be as high as 86–90% in displaced injuries, especially in younger patients with substantial remaining growth potential [1,5].

We present a case of an open Salter–Harris type I distal femoral physeal fracture in a 6-year-old child complicated by progressive growth arrest and significant limb-length discrepancy despite appropriate initial management. This case highlights the importance of long-term follow-up and growth prediction in managing these injuries. A focused review of the literature is also provided to contextualize the findings and discuss current treatment strategies.

## 2. Case Report

### 2.1. Initial Examination

A previously healthy 6-year-old child with no relevant past medical or surgical history was admitted to the emergency department following a high-energy electric scooter–motor vehicle collision. On arrival, a structured primary assessment was performed according to Advanced Trauma Life Support (ATLS) principles.

The patient was brought to the emergency department with a pre-hospital pelvic binder and a combat application tourniquet (CAT) applied to the left lower limb. The patient was hemodynamically unstable, with clinical signs of hemorrhagic shock due to active bleeding from the injured limb. The Glasgow Coma Scale score was 15, and no signs of traumatic brain injury were identified.

**Figure 1:**
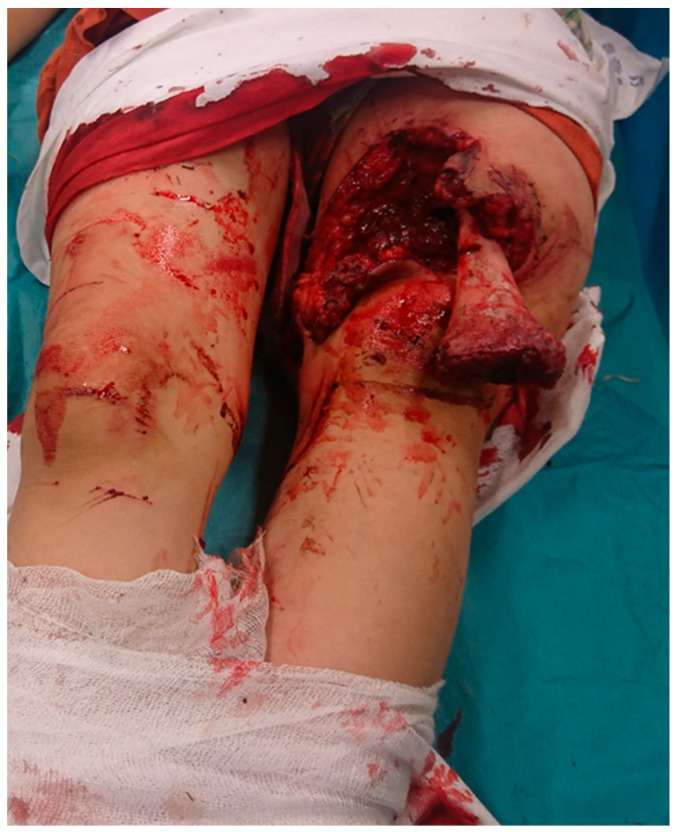
Clinical appearance of the left distal thigh on admission, demonstrating severe deformity and a large open soft-tissue wound.

Physical examination revealed a gross deformity of the left distal thigh associated with a large open laceration measuring approximately 20–25 cm, active bleeding, and extensive soft-tissue disruption (Figure 1). Distal perfusion was preserved, with palpable dorsalis pedis and posterior tibial pulses, normal capillary refill, and no evidence of acute vascular compromise. Neurological examination of the limb was unremarkable.

Secondary survey identified only superficial pelvic abrasions, with no clinical evidence of thoracic or abdominal injury.

Initial laboratory evaluation revealed severe acute blood loss, with a hemoglobin level of 69 g/L. To restore the circulating blood volume, a single unit (275 mL) of packed red blood cells was transfused. Following the red blood cell transfusion, the hemoglobin levels stabilized at 119 g/L, indicating effective correction of the acute anemia; no further blood products were required. Analysis of the post-transfusion urinalysis revealed significant glucosuria (56 mmol/L) and microscopic erythrocyturia (25 cells/μL).

### 2.2. Assessment and Diagnosis

Focused Assessment with Sonography for Trauma (FAST) showed no intra-abdominal free fluid, and chest radiography demonstrated no acute thoracic pathology.

Radiographic evaluation of the left femur demonstrated a displaced distal femoral fracture involving the physeal region. Due to the patient’s unstable condition and the severity of the open injury, definitive classification was established intraoperatively.

Based on intraoperative findings—complete fracture of the femur across the entire epiphyseal-physeal junction with separation; the injury was classified as a Salter–Harris type I distal femoral physeal fracture.

### 2.3. Operative Management

Given the open nature of the injury, extensive soft-tissue damage, and ongoing hemorrhage, urgent operative intervention was undertaken. Intravenous cefazolin was administered upon admission.

**Figure 2:**
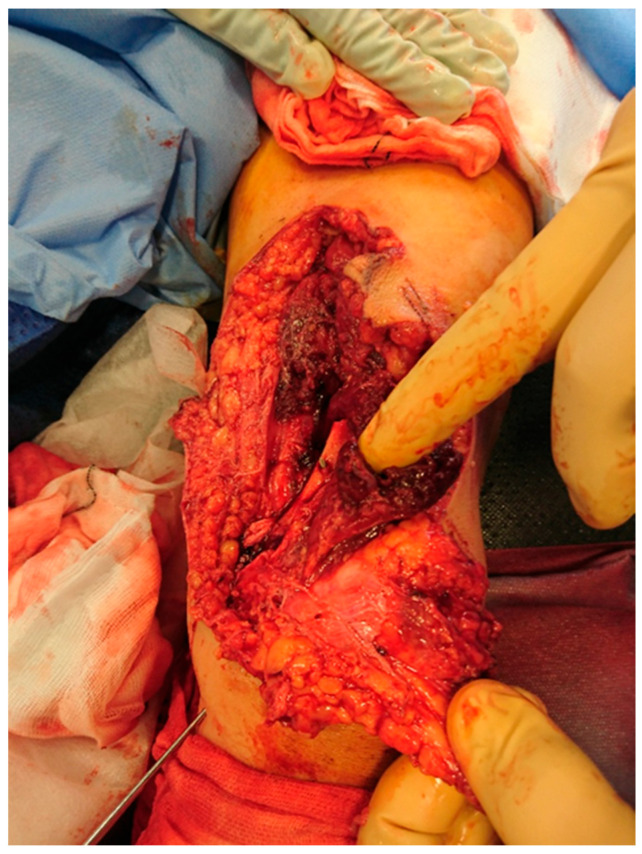
Intraoperative debridement of the open distal femoral injury before definitive stabilization.

**Figure 3:**
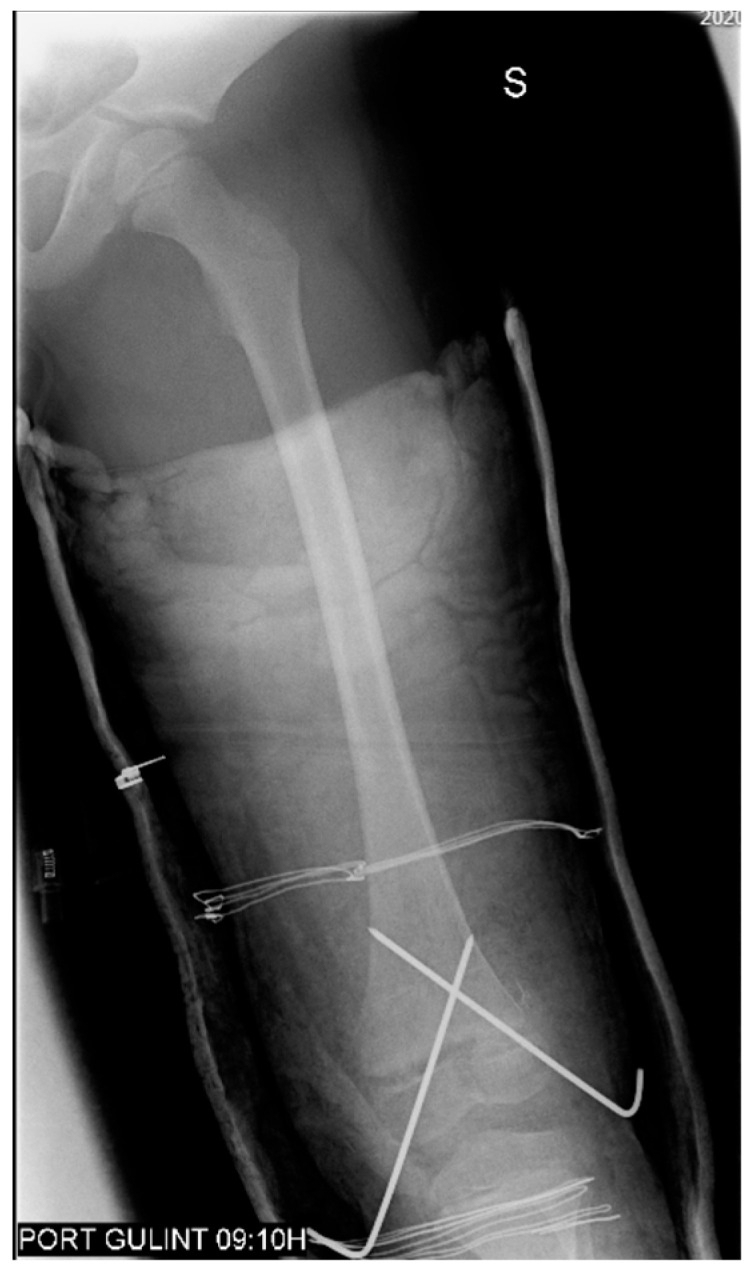
Postoperative anteroposterior radiograph demonstrating crossed smooth Kirschner-wire fixation of the distal femoral physeal fracture in a cast mobilization.

In the operating room, the wound underwent thorough irrigation and meticulous debridement (Figure 2). Open reduction of the distal femoral physeal fracture was performed, followed by stabilization using two crossed smooth 1.6 mm Kirschner wires inserted across the physis to achieve adequate fixation. Following layered closure of the wound and the insertion of an active suction drain, peripheral perfusion was assessed. The posterior tibial artery pulse was palpable, reflecting adequate peripheral perfusion without signs of ischemia. The limb was immobilized in a posterior long-leg splint (Figure 3).

As the child was fully immunized, no additional tetanus prophylaxis was required. Postoperatively, the patient received a 14-day course of intravenous cefazolin (1 g, three times daily) and 5 days of anticoagulant therapy with nadroparine (Fraxiparine, 0.3 mL daily). The drain was removed on the second postoperative day. While the posterior wound healed by primary intention, the anterior thigh wound exhibited localized skin necrosis. On the fifth day, purulent discharge was observed, necessitating the insertion of Penrose drains. Notably, the microbiological cultures obtained from the left thigh wound yielded no bacterial growth. One week after the initial procedure, a second operation was performed for repeat debridement and excision of necrotic tissue.

### 2.4. Follow-Up

**Figure 4:**
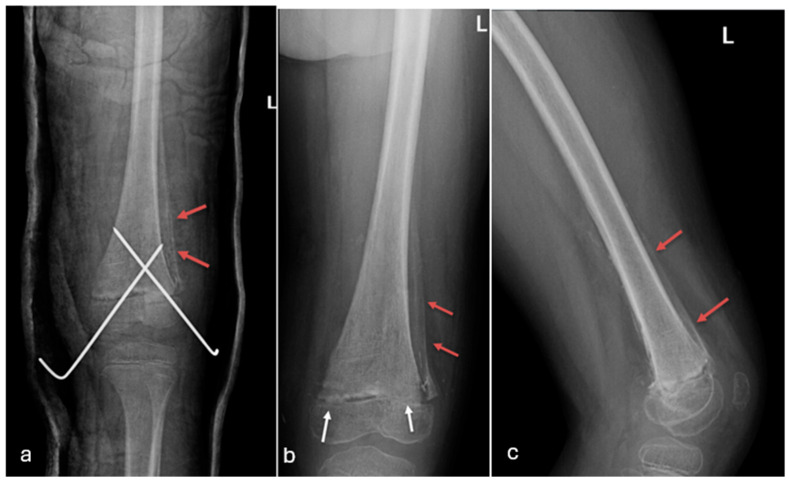
X-ray evaluation: anteroposterior radiograph: (**a**) one month after the operation, evaluation regarding the stability of fragments, and consolidation is underway; (**b**) Anteroposterior radiograph two months after operation, morphological irregularities (closing growth plate) were noted within the distal femoral physis (white arrows), as well as revealing signs of periosteal proliferation, likely secondary to the ossification of a post-traumatic soft tissue hematoma (red arrows); (**c**) Lateral radiograph of the evaluation of the femoral diaphysis.

**Figure 5:**
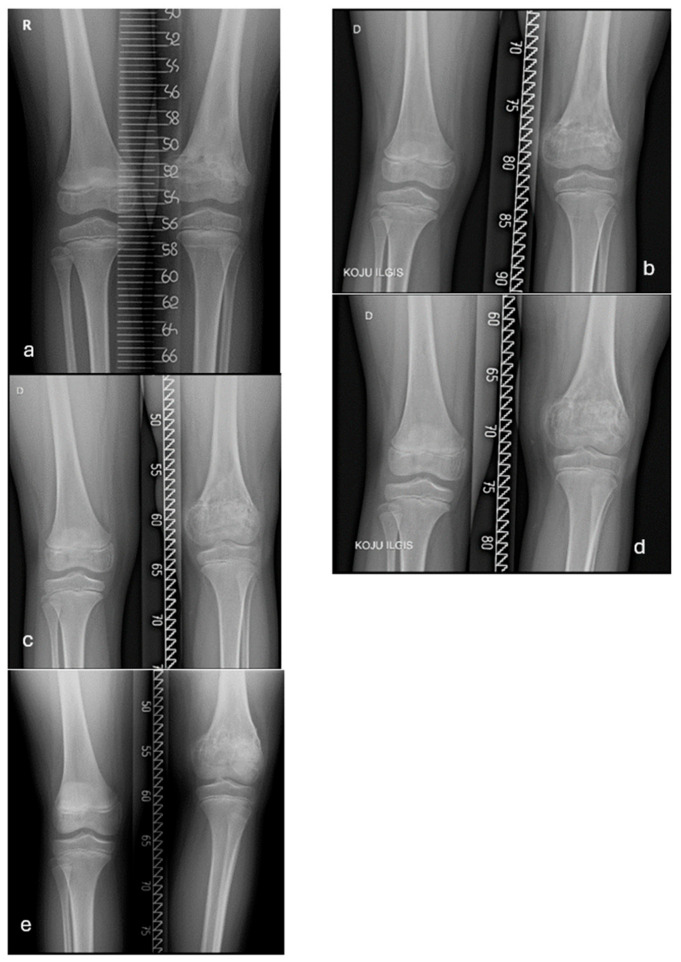
Long-term outpatient follow-up and dynamic assessment of the patient’s growth, anteroposterior radiograph. (**a**) 10 months post-injury, limb-length discrepancy of 1.3 cm. (**b**) 18 months post-injury, limb-length discrepancy of 2.3 cm. (**c**) 36 months post-injury, limb-length discrepancy of 4.1 cm. (**d**) 3.5 years post-injury, limb-length discrepancy of 2.3 cm. (**e**) 5 years post-injury, limb-length discrepancy of 6.5 cm.

The patient was followed up at 2 weeks, 1 month, 2 months, 10 months, 18 months, 2 years, 3.5 years, and 5 years postoperatively (Figure 4 and Figure 5).

Clinical examination at 1 month postoperatively demonstrated complete soft-tissue healing. X-ray evaluation revealed stable fragment alignment and a bridging callus, indicating active consolidation (Figure 4). Following these findings, the Kirschner wires were subsequently removed in an outpatient setting.

Two months postoperatively, the patient was readmitted for planned staged management, including hardware extraction (Figure 4). Clinical examination at the time of admission demonstrated a restricted range of motion (contracture) in the left knee and significant quadriceps muscle weakness. Neurological assessment showed symmetrical deep tendon reflexes (dextra = sinistra). The patient remained non-ambulatory and was unable to tolerate axial weight-bearing on the affected limb. Serial radiographs confirmed persistent morphological changes in the distal femoral growth plate.

**Figure 6:**
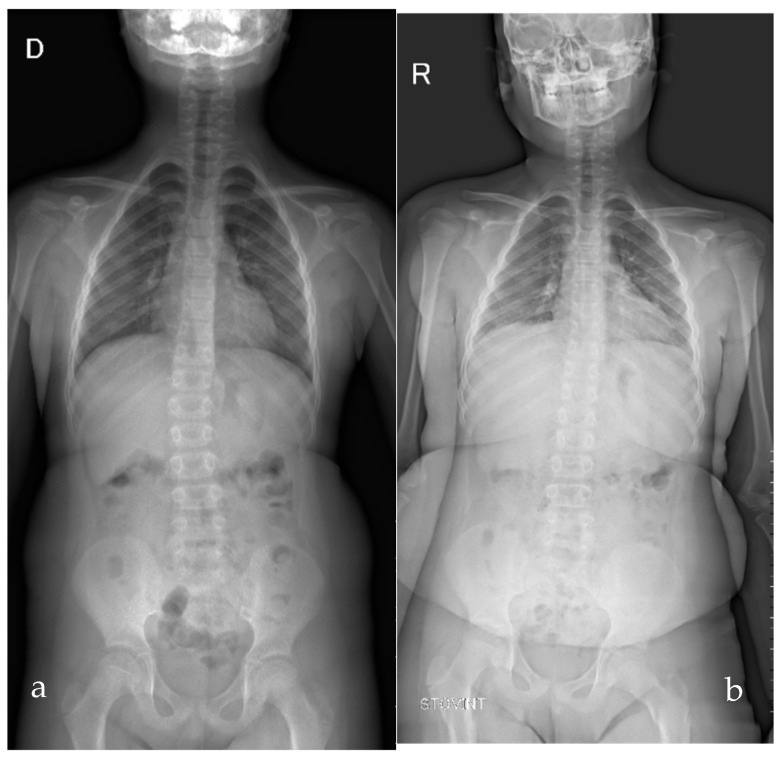
Full-length spinal radiograph, posteroanterior view. (**a**) 3.5 years post-injury, radiographic assessment revealed a minor thoracolumbar dextroscoliosis measuring ~9° according to the Cobb method. (**b**) 5 years post-injury, no significant progression of the scoliosis was observed.

At 10 months post-injury, radiographic evaluation demonstrated post-traumatic changes of the distal femur and a limb-length discrepancy of 1.3 cm (Figure 5). During the subsequent 5 years of follow-up, progressive and uniform shortening of the injured limb was observed, with the discrepancy increasing to 6.5 cm. No significant angular deformity of the limbs was noted (Figure 5). However, a slight curvature of the spine with a ~9° Cobb angle was observed (Figure 6). Due to the significant leg-length discrepancy, the patient was prescribed custom-made complex orthopedic footwear with a 5 cm heel lift.

**Figure 7:**
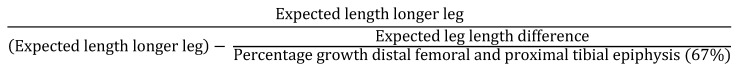
Multiplier-method calculation used to estimate predicted final limb-length discrepancy and timing of contralateral epiphysiodesis.

Growth prediction using the multiplier method estimated final limb lengths of 87.84 cm for the contralateral limb and 80.52 cm for the affected limb, corresponding to a predicted discrepancy of 7.32 cm at skeletal maturity (Figure 7). Based on these calculations, the optimal timing for surgical intervention was estimated at approximately 12.5–13 years of age. The percentage growth left in the longer (right) leg at the time of epiphysiodesis is 14.2%. To verify this percentage, the difference between the expected leg length and the post-intervention length is 10.93 cm. This represents 14.2% of the remaining total growth, of which 67% is attributed by the distal femoral and proximal tibial epiphysis.

Six years after the initial injury, contralateral distal femoral epiphysiodesis was performed to address the projected discrepancy. During the surgical intervention, skin incisions on the right limb were made over the distal femoral and proximal tibial physis. Permanent epiphysiodesis was achieved through mechanical ablation using a drill and curettes. The post-operative time was uneventful.

Follow-up is ongoing, and staged limb lengthening of the affected limb remains a potential future option depending on clinical progression.

Written informed consent for publication of the case details and accompanying images was obtained from the patient’s parents.

## 3. Discussion

Distal femoral physeal fractures are uncommon injuries, accounting for approximately 0.3–1.4% of all pediatric fractures; however, they are associated with a high rate of significant complications, including premature physeal closure and subsequent growth disturbance [1,2]. The distal femoral physis contributes substantially to longitudinal growth of the femur and lower limb, which explains the clinical importance of injuries at this site [3]. These fractures are more commonly reported in older children and adolescents and are usually associated with high-energy trauma [4,5]. Bellamy et al. reported a mean age of 12.5 years and found that growth disturbances were more frequent in younger patients [5]. Open femoral fractures are rare in the pediatric population, accounting for up to 3% of femoral fractures [6]. In this context, the present case is unusual because of the patient’s young age, the open nature of the injury, and the later development of marked limb-length discrepancy.

Distal femoral physeal fractures most commonly result from high-energy mechanisms, including motor vehicle collisions, falls from height, and sports-related trauma [3,7]. Because the surrounding ligamentous structures are often stronger than the physis, traumatic forces may preferentially injure the growth plate rather than produce ligament rupture [3]. The distal femoral physis is responsible for approximately 70% of femoral growth and 37% of total lower-limb length [3]; therefore, physeal damage at this level may lead to premature closure, angular deformity, and limb-length discrepancy. Long-term outcome is influenced by the severity of the initial physeal insult, fracture displacement, patient age, and associated soft-tissue injury [1,5,8]. In the present case, the injury resulted from an e-scooter-versus-motor vehicle collision and was accompanied by extensive soft-tissue damage, both of which likely increased the risk of later growth arrest. It demonstrates that, following an injury involving significant kinetic force at a young age, continuous leg-length monitoring is essential to detect potential growth plate damage and subsequent growth disturbances.

Plain radiography remains the first-line imaging modality for distal femoral physeal fractures, allowing assessment of fracture pattern and displacement [6,7,9]. CT or MRI may be useful in selected cases, particularly when radiographs are inconclusive or when further evaluation of physeal or articular injury is required [9,10]. In the present case, complete imaging assessment in the acute setting was limited because urgent operative management took priority owing to hemodynamic instability and the severity of the open injury.

Treatment should be individualized according to fracture type, displacement, patient age, and the extent of soft-tissue damage. Stable non-displaced fractures may be treated conservatively, whereas displaced or unstable injuries generally require reduction, fixation, and immobilization [2,3,7,9]. Smooth Kirschner wires may be passed across the physis when necessary to achieve stable fixation, and available evidence suggests that later growth disturbance is more strongly related to the initial injury than to transphyseal wire placement itself [3]. For young children, stabilization with Kirschner wires is usually the first-line treatment [11]. A retrospective study found that removing an impacted periosteum does not reduce the risk of premature physeal closure [5]. In the present case, fixation with two smooth crossed 1.6 mm Kirschner wires provided initial stability, but did not prevent progressive growth disturbance during follow-up. This approach was used due to the child’s young age, the small bone diameter, and the narrow medullary canal.

The prognosis of distal femoral physeal fractures depends on patient age, fracture displacement, injury severity, and associated soft-tissue damage [1,5,12]. Because of the major contribution of the distal femoral physis to lower-extremity growth, these injuries carry a particularly high risk of premature physeal closure and subsequent limb-length discrepancy or angular deformity [3]. Recent evidence suggests that in young patients with significantly displaced Salter–Harris type I and II distal femoral fractures, the risk of premature physeal closure may be as high as 90% [12]. A retrospective study found a significant correlation: the younger the patient (i.e., the more years remaining until skeletal maturity), the greater the likelihood of additional growth-correction surgeries [5]. Moreover, a significant fracture displacement (>50%) increases the probability of growth plate closure to 73% [12]. This indicates that prognosis is determined not only by fracture classification, but also by the severity of the initial trauma and the amount of remaining growth.

When progressive unilateral limb-length discrepancy develops, contralateral epiphysiodesis may be considered to reduce the final discrepancy [13]. Accurate timing is essential and should be based on skeletal maturity and growth-prediction methods [13,14,15].

In the present case, limb-length discrepancy increased to 6.5 cm over five years, requiring precise growth prediction to determine the optimal timing for epiphysiodesis. Recent literature indicates that the timing of surgical treatment for acquired leg length discrepancies can be determined without resorting to older, more complex methods, such as the Moseley straight-line graph, but rather using simplified Paley’s multiplier methods [13]. The most important aspect of applying the multiplier is consistent measurement of the patient’s leg. In our clinical case, the patient was monitored annually, and leg length discrepancy was calculated. The current proportional relationship between the growth rates of the healthy and affected legs ensures a reliable prognosis for determining the appropriate timing of surgery. The multiplier method predicted a final discrepancy of 7.32 cm without surgery. These findings supported contralateral distal femoral epiphysiodesis as an appropriate growth-modulating procedure.

This clinical case presents a significant prognostic challenge due to the early onset of trauma, resulting in the progressive limb length discrepancy in a patient with substantial remaining growth potential. A case poses medical challenges in selecting the optimal timing for epiphysiodesis to ensure that the remaining growth potential is fully utilized, maximizing correction of the existing discrepancy without causing overcorrection or residual discrepancy. Due to the risk of angular deformity—the most frequent postoperative complication—patients must be monitored until skeletal maturity. In a retrospective study, valgus alignment was the most common deformity after epiphysiodesis [15]. Even for epiphysiodesis being the gold standard for discrepancy treatment, in this case, epiphysiodesis alone is insufficient due to a continuing significant difference in leg length. A staged combined treatment strategy is employed, combining growth modulation with distractive intramedullary limb lengthening at skeletal maturity. Gradual lengthening preserves soft tissue integrity. Intramedullary nails, used for leg lengthening, significantly lower the infection risks compared to external methods [14,16]. Final limb length equalization in our case will be managed via intramedullary distraction, aiming for a target discrepancy of 1.0 to 1.5 cm.

This case therefore, underscores the need for prolonged follow-up after distal femoral physeal injury, even when early management is timely and technically adequate.

## 4. Conclusions

Distal femoral physeal fractures caused by high-energy trauma carry a substantial risk of premature physeal closure and progressive limb-length discrepancy, particularly in younger children. This case highlights the importance of urgent management and prolonged follow-up, as growth disturbance may develop despite appropriate initial treatment. Growth-prediction methods are useful for planning timely corrective intervention.

## Data Availability

All data generated or analyzed during this study are included in this published article. Further inquiries can be directed to the corresponding author.
